# Differences Between HIV-Infected Men and Women in Antiretroviral Therapy Outcomes — Six African Countries, 2004–2012

**Published:** 2013-11-29

**Authors:** Virginie Ettiègne-Traoré, Moise Zanga Tuho, Fayama Mohamed, Charles Azih, Francisco Mbofana, Modest Mulenga, Fred Wabwire-Mangen, Gideon Kwesigabo, Joseph Essombo, Harrison Kamiru, Harriet Nuwagaba-Biribonwoha, Kwasi Torpey, Eric Van Praag, Ya Diul Mukadi, Olivier Koole, Joris Menten, Robert Colebunders, Lisa J. Nelson, Georgette Adjorlolo-Johnson, Julie Denison, Sharon Tsui, Carol Dukes Hamilton, Timothy Mastro, David Bangsberg, Kunomboa A. Ekra, Joseph S. Kouakou, Peter Ehrenkranz, Trong Ao, Charity Alfredo, Kebba Jobarteh, Seymour Williams, Ray W. Shiraishi, Andrew Baughman, Simon Agolory, George Bicego, Thomas Spira, Aaron Zee, Jonathan Kaplan, Tedd V. Ellerbrock, Andrew F. Auld

**Affiliations:** Ministry of Health; Directorate General of Budget and Finance, Côte d’Ivoire; Ministry of Health, Swaziland; National Institute of Health, Mozambique; Tropical Diseases Research Center, Zambia; The Infectious Diseases Institute, Makerere Univ College of Health Sciences, Uganda; Muhimbili Univ of Health and Allied Sciences, Tanzania; Elizabeth Glaser Pediatric AIDS Foundation, Côte d’Ivoire; International Center for AIDS Care and Treatment Programs-Columbia Univ, Swaziland; FHI 360, Zambia; FHI 360, Tanzania; FHI 360, Haiti; Institute of Tropical Medicine, Dept of Clinical Sciences, Belgium; Dept of HIV/AIDS, World Health Organization, Switzerland; Elizabeth Glaser Pediatric AIDS Foundation, California; Social and Behavioral Health Sciences, FHI 360, Washington, DC; Global Health, Population & Nutrition, FHI 360, Durham, North Carolina; Massachusetts General Hospital; Center for Global Health, CDC-Côte d’Ivoire; Center for Global Health, CDC-Swaziland; Center for Global Health, CDC-Mozambique; Center for Global Health, CDC-South Africa; Div of Global HIV/AIDS, Center for Global Health, CDC

Evaluation of differences between human immunodeficiency virus (HIV)-infected men and women in antiretroviral therapy (ART) enrollment characteristics and outcomes might identify opportunities to improve ART program patient outcomes and prevention impact. During September 2008–February 2012, retrospective cohort studies to estimate attrition of enrollees (i.e., from death, stopping ART, or loss to follow-up) at 6-month intervals after ART initiation were completed among samples of adult men and women (defined as aged ≥15 years or aged ≥18 years) who initiated ART during 2004–2010 in six African countries: Côte d’Ivoire in western Africa; Swaziland, Mozambique, and Zambia in southern Africa; and Uganda and Tanzania in eastern Africa. Records for 13,175 ART enrollees were analyzed; sample sizes among the six countries ranged from 1,457 to 3,682. In each country, women comprised 61%–67% of ART enrollees. Median CD4 count range was 119–141 cells/*μ*L for men and 137–161 cells/*μ*L for women. Compared with women, a greater percentage of men initiated ART who had World Health Organization (WHO) HIV stage IV disease. In cohorts from western Africa and southern Africa, the risk for attrition was 15%–26% lower among women compared with men in multivariable analysis. However, in eastern Africa, differences between men and women in risk for attrition were not statistically significant. Research to identify country-specific causes for increased attrition and delayed initiation of care among men could identify strategies to improve ART program outcomes among men, which might contribute to prevention of new HIV infections in female partners.

In each of the six countries, a representative sample of ART facilities was selected. To keep the studies feasible, small facilities were excluded from the sample frames of countries with >100 ART facilities at the time of sampling. Therefore, in Côte d’Ivoire and Mozambique, facilities that had enrolled <50 adults on ART were excluded, whereas in Zambia, Uganda, and Tanzania, facilities that had enrolled <300 adults on ART were excluded (Table 1).

From the eligible number of facilities in Côte d’Ivoire (78), Swaziland (31), and Mozambique (94), totals of 34, 16, and 30 study facilities, respectively, were randomly selected using probability-proportional-to-size sampling (Table 1). From the eligible number of facilities in Zambia (129), Uganda (114), and Tanzania (85), six study facilities were purposefully (nonrandomly) selected in each country to represent different types of ART facilities.

At each selected facility, a sample frame of study-eligible ART patients was created, and simple random sampling used to select the desired sample size of patient medical records. Eligibility criteria included having started ART during 2004–2010 and ≥6 months before data abstraction. Data were abstracted by trained study personnel from ART medical records onto standardized abstraction forms.

Attrition was the primary outcome of interest. A patient was considered lost to attrition if the record showed 1) the patient had died, 2) the patient had stopped ART because of a personal or clinician decision, or 3) the patient had not attended the facility in the 90 days preceding data abstraction for either medication refill or a clinician visit, in which case the patient was considered lost to follow-up.

Variables routinely collected on Ministry of Health medical records at ART initiation including sex, age, CD4 count, WHO HIV disease stage, and ART regimen, were entered into standardized abstraction forms. Data were analyzed using statistical software, and study design was controlled for during analysis. Data for Côte d’Ivoire, Swaziland, and Mozambique were weighted to account for the probability-proportional-to-size sampling.

To estimate the effect of sex on attrition, Cox proportional hazards regression models were used to estimate unadjusted and adjusted hazard ratios, 95% confidence intervals, and p-values. Multivariate models included only cases with complete data for sex, age, CD4 count, WHO HIV disease stage, and ART regimen. The proportional hazards assumption was assessed using visual methods and the Grambsch and Therneu test (1). For all countries, a shared frailty model was used to account for intrafacility correlation. Chi-square tests were used to compare distributions of categorical variables, and t-tests were used to compare distributions of continuous variables between men and women. Attrition at 6-month intervals after ART initiation was analyzed using the Kaplan-Meier product-limit estimate of the survivor function.

The sample sizes in the six countries ranged from 1,457 to 3,682, with a total of 13,175 records analyzed (Table 2). In each country, 61%–67% of ART enrollees were women. In each country, men were significantly older than women at ART initiation. Median age range for men was 37–40 years, and for women was 32–35 years.

Compared with women, median CD4 count at ART initiation was significantly lower among men in Côte d’Ivoire (119/*μ*L compared with 155/*μ*L), Swaziland (121/*μ*L compared with 161/*μ*L), and Uganda (128/*μ*L compared with 144/*μ*L). Median CD4 count was lower for men compared with women in Mozambique, Zambia, and Tanzania, although these differences were not statistically significant (p>0.05).

In all countries, men initiated ART at a more advanced WHO disease stage (Table 2). For example, a higher proportion of men than women initiated ART at WHO stage IV disease in Côte d’Ivoire (26% compared with 21%), Swaziland (18% compared with 10%), Mozambique (20% compared with 13%), Zambia (11% compared with 9%), Uganda (16% compared with 10%), and Tanzania (30% compared with 27%).

For both men and women across all six countries, nevirapine-containing first-line regimens were more common than efavirenz-containing or protease inhibitor–containing regimens, or triple nucleoside reverse transcriptase inhibitor regimens (Table 2). The distribution of first-line ART regimen choices for men differed from choices for women in all countries, with women more likely to be prescribed nevirapine-containing regimens than men (Table 2).

In all countries, point estimates for attrition during the first 4.5 years of ART were lower for women than men (Table 3). This difference was statistically significant in countries in western and southern Africa, where women had 21%–27% lower rates of attrition in unadjusted analysis and 15%–26% lower rates of attrition in adjusted analysis (Table 3). In Uganda and Tanzania, women had 7%–18% lower rates of attrition in unadjusted analysis and 11%–12% lower rates of attrition in adjusted analysis, although the associations between sex and attrition rates were not statistically significant in these two countries.

## Editorial Note

Equitable access to ART for both men and women is a principle endorsed by most African governments and international donors, including the U.S. President’s Emergency plan for AIDS Relief and the Global Fund to Fight HIV/AIDS, Tuberculosis, and Malaria (2,3). However, in Africa, proportionally more HIV-infected women are accessing ART services than men (4). Evaluating differences in ART enrollment characteristics and treatment outcomes between men and women might help program managers understand these differences in ART enrollment and identify opportunities for ART program improvement.

This report has two main findings: 1) among representative samples of adult ART enrollees in six African countries, men were more likely than women to initiate ART with advanced HIV disease, and 2) men had higher attrition risk than women after ART initiation. However, differences between men and women in rate of attrition varied by country, being larger and statistically significant in western and southern African cohorts, but smaller and not statistically significant in cohorts from eastern Africa (Uganda and Tanzania).

As in other reports from African ART programs (2,5–7), men initiated ART at more advanced HIV disease stages than women. Late initiation of ART among men has commonly been attributed to sex differences in health-seeking behavior, with men considered more likely to delay access to health care for various reasons, including stigma, male norms that discourage admitting ill health, and employment responsibilities (2). However, some have proposed that the prioritization of maternal and child health services by global and national public health organizations in Africa has resulted in inequitable access to health services, including ART (2,3). Recent reports suggest that whereas nearly all African countries include initiatives focused on women in their national AIDS strategies, only 10% of countries are effectively engaging men and boys in the national AIDS response (8). To address delayed enrollment in ART among men, increased attention from national governments and international donors to identify and implement evidence-based strategies that achieve earlier HIV testing and ART among men might be needed (2,3).

As in other studies from western and southern Africa (2,5,6,9), adjusting for possible baseline predictors of ART outcomes, including CD4 count, WHO HIV disease stage, age, and ART regimen, did not fully account for the increased rate of attrition among men in Côte d’Ivoire, Swaziland, Mozambique, and Zambia. This suggests unmeasured factors are contributing to either increased rates of death or loss to follow-up among men (2). Some reports have suggested sex differences in health-seeking behavior, biologic differences in response to ART, increased male risk for opportunistic infections, worse adherence to ART pill-taking among men, or background differences in mortality rates by sex in the general population are responsible for higher attrition rates among men taking ART (2).

In this analysis, although rates of attrition were marginally higher among men than women in Uganda and Tanzania, the effect of sex on attrition risk was smaller than that observed in cohorts from western and southern Africa. This might indicate variations in the effect of sex on attrition risk by country or region. One possible explanation is that internal and cross-border migration patterns vary by country and region. Historically, cross-border migration for work, which is more common among men than women, varies by region in Africa, being most common in western (10) and southern Africa, where South Africa is a hub for migrant labor from surrounding countries (10). Further research into variations in the effect of sex on attrition risk by country or region might inform interventions to reduce rates of male attrition.

The findings in this report are subject to at least two limitations. First, missing data for certain covariates of interest at ART initiation might have introduced some measurement error, and might have affected estimates of hazard ratios. Second, because of differences in cohort size, there was greater power to detect differences in outcomes between men and women in Swaziland, Mozambique, and Côte d’Ivoire than in Zambia, Uganda, and Tanzania, which limits ability to make conclusions about country variations in the effect of sex on attrition risk.

Across six countries in Africa, men initiated ART with more advanced disease and had statistically significant higher attrition in western and southern African cohorts. Reasons for differences between men and women in ART enrollment are not fully understood but might include lack of emphasis by donors and national governments on the importance of engaging men early in ART (3). Higher attrition rates among men are not fully explained by traditional predictors of poor outcomes (e.g., low CD4 count and advanced WHO HIV disease stage). Identifying and implementing evidence-based interventions to improve male enrollment and retention in ART programs is important to reduce male AIDS-related mortality and might contribute to prevention of new HIV infections in female partners (3).

What is already known on this topic?

Evaluating differences between human immunodeficiency virus (HIV)-infected men and women in antiretroviral therapy (ART) enrollment characteristics and treatment outcomes can help program managers understand why proportionally more women than men are accessing ART.

What is added by this report?

This retrospective cohort study of six African countries found lower median CD4 counts and more World Health Organization stage IV HIV disease in men at enrollment in all six countries. In addition, the risk of attrition during ART was significantly higher in men in western and southern African countries, even after controlling for possible baseline predictors of ART outcomes. This finding suggests that unidentified factors are contributing to this higher attrition risk in these countries. In eastern Africa, risk for attrition did not differ significantly between men and women.

What are the implications for public health practice?

Further research on country-specific reasons for differences between HIV-infected men and women in ART enrollment and in attrition while on ART are needed. The results of such studies could potentially identify strategies to improve early diagnosis and treatment among men and improve program outcomes.

## Figures and Tables

**TABLE 1:** Summary of sampling criteria and methods*† used to select adult antiretroviral therapy (ART) enrollees for retrospective cohort studies conducted — six African countries, 2008–2012

Characteristic	Côte d’Ivoire	Swaziland	Mozambique	Zambia	Uganda	Tanzania	Total
**Stage 1: Selection of study facilities**							
No. of ART clinics	124 by Dec 2007	31 by Dec 2009	152 by Dec 2006	322 by Dec 2007	286 by Dec 2007	210 by Dec 2007	
No. of enrollees at ART clinics	36,943	50,767	43,295	65,383	45,946	41,920	
Clinic eligibility criteria for study	Enrolled ≥50 adults on ART by Dec 2007	All ART initiation sites eligible	Enrolled ≥50 adults on ART by Dec 2006	Enrolled ≥300 adults on ART by Dec 2007	Enrolled ≥300 adults on ART by Dec 2007	Enrolled ≥300 adults on ART by Dec 2007	
No. of study-eligible clinics	78	31	94	129	114	85	**531**
Estimated no. of study-eligible enrollees at clinics	36,110	50,767	42,234	58,845§	41,351§	37,728§	**267,035**
No. of clinics selected	34	16	30	6	6	6	**98**
**Stage 2: Selection of study patients**							
Age at ART initiation criteria for study enrollees	≥15 yrs	≥15 yrs	≥15 yrs	≥18 yrs	≥18 yrs	≥18 yrs	
Years of ART enrollment	2004–2007	2004–2010	2004–2007	2004–2009	2004–2009	2004–2009	
Planned sample size	4,000	2,500	2,600	1,500	1,500	1,500	**13,600**
No. of eligible study enrollees	3,682	2,510	2,596	1,457	1,472	1,458	**13,175**
Date of data collection	Nov 2009–March 2010	Nov 2011–Feb 2012	Sept–Nov 2008	April–July 2010	April–July 2010	April–July 2010	

* In Côte d’Ivoire, Swaziland, and Mozambique, study facilities were randomly selected using probability-proportional-to-size sampling. In Zambia, Uganda, and Tanzania, study facilities were purposefully selected to represent different types of ART facilities in each country.

† In all six countries, at each selected facility, a sample frame of study-eligible ART patients was created, and simple random sampling was used to select the desired sample size of patient medical records.

§ Estimate from available published data.

**TABLE 2:** Enrollment characteristics of adults (N = 13,175) initiating antiretroviral therapy (ART) — six African countries, 2004–2010

	Côte d’Ivoire* (N = 3,682)			Swaziland* (N = 2,510)			Mozambique* (N = 2,596)			Zambia† (N = 1,457)			Uganda† (N = 1,472)			Tanzania† (N = 1,458)		
						
Characteristic	No.	Median age	p-value§	No.	Median age	p-value	No.	Median age	p-value	No.	Median age	p-value	No.	Median age	p-value	No.	Median age	p-value
**Sex, no., and median age**																		
Women	2,422	34	<0.001	1,621	32	<0.001	1,576	32	<0.001	880	34	<0.001	964	34	<0.001	973	35	<0.001
Men	1,260	40		889	38		1,020	38		575	37		502	37		484	39	
	**No.**	**Median CD4**	**p-value**	**No.**	**Median CD4**	**p-value**	**No.**	**Median CD4**	**p-value**	**No.**	**Median CD4**	**p-value**	**No.**	**Median CD4**	**p-value**	**No.**	**Median CD4**	**p-value**

**Sex, no., and median CD4 count at ART initiation (cells/** *μ* **L)**																		
Women	1,811	155	<0.001	1,487	161	<0.001	1,373	161	0.063	639	139	0.261	774	144	0.012	742	137	0.232
Men	935	119		809	121		881	141		411	127		395	128		379	121	
Missing data	936	—		214	—		342	—		407	—		303	—		337	—	
	**No.**	**(%)**	**p-value**	**No.**	**(%)**	**p-value**	**No.**	**(%)**	**p-value**	**No.**	**(%)**	**p-value**	**No.**	**(%)**	**p-value**	**No.**	**(%)**	**p-value**

**Sex, no., and %**																		
Women	2,422	(67)		1,621	(65)		1,576	(62)		882	(61)		968	(66)		974	(67)	
Men	1,260	(33)		889	(35)		1,020	(38)		575	(39)		504	(34)		484	(33)	
**WHO HIV disease stage**																		
Stage I/II																		
Women	363	(19)	0.064	700	(50)	<0.001	386	(39)	0.001	326	(41)	0.029	434	(51)	0.006	225	(29)	0.006
Men	224	(22)		260	(34)		233	(34)		170	(34)		205	(45)		76	(20)	
Stage III																		
Women	987	(61)		590	(40)		451	(48)		390	(49)		330	(39)		347	(44)	
Men	453	(53)		377	(48)		288	(46)		277	(55)		178	(39)		192	(50)	
Stage IV																		
Women	331	(21)		149	(10)		136	(13)		73	(9)		87	(10)		212	(27)	
Men	223	(26)		144	(18)		123	(20)		54	(11)		73	(16)		113	(30)	
Missing data	1,101	(30)		290	(12)		979	(38)		167	(11)		165	(11)		293	(20)	
**Female regimens**																		
NVP-3TC-D4T/AZT/TDF	1,219	(49)	0.077	1,153	(72)	<0.001	1,430	(91)	<0.001	606	(69)	<0.001	772	(80)	0.013	760	(78)	0.088
EFV-3TC-D4T/AZT/TDF	692	(30)		372	(23)		124	(8)		260	(29)		179	(18)		201	(21)	
Triple NRTI	90	(3)		0	—		13	(1)		0	—		3	(<1)		0	—	
PI-based	122	(4)		0	—		1	(<1)		6	(1)		8	(1)		0	—	
Unknown	211	(10)		96	(5)		8	(<1)		0	—		0	—		0	—	
Other	88	(5)		0	—		0	—		10	(1)		6	(1)		13	(1)	
**Male regimens**																		
NVP-3TC-D4T/AZT/TDF	548	(42)		519	(59)		881	(85)		334	(58)		362	(72)		357	(74)	
EFV-3TC-D4T/AZT/TDF	430	(36)		309	(35)		120	(13)		225	(39)		133	(26)		122	(25)	
Triple NRTI	48	(3)		0	—		4	(<1)		0	—		1	(<1)		0	—	
PI-based	84	(6)		1	(<1)		2	(<1)		9	(2)		5	(1)		1	(<1)	
Unknown	110	(9)		60	(6)		13	(1)		0	—		0	—		0	—	
Other	40	(3)		0	—		0	—		7	(1)		3	(<1)		974	(1)	

**Abbreviations:** WHO = World Health Organization; NVP = nevirapine; EFV = efavirenz; 3TC = lamivudine; D4T = stavudine; AZT = zidovudine; TDF = tenofovir; NRTI = nucleoside reverse transcriptase inhibitor; PI = protease inhibitor.

* Data for Côte d’Ivoire, Swaziland, and Mozambique are weighted to account for the sampling method.

† Data were missing for two enrollees in Zambia, six in Uganda, and one in Tanzania.

§ p-value for comparison of ART enrollee characteristics for men and women (t-test for continuous variables and chi-square test for categorical variables).

**TABLE 3:** Percentage of antiretroviral therapy (ART) enrollees alive and on therapy, by years after ART initiation, and attrition* rate and risk for attrition, by sex — retrospective cohort studies, six African countries

	Côte d’Ivoire			Swaziland			Mozambique			Zambia			Uganda			Tanzania		
						
Years after ART initiation	Women %	Men %		Women %	Men %		Women %	Men %		Women %	Men %		Women %	Men %		Women %	Men %	
0.5	81	75		86	81		84	81		81	75		94	91		76	75	
1	76	70		82	77		78	73		77	69		90	88		71	67	
1.5	72	66		79	74		74	69		73	65		87	84		65	62	
2	67	60		76	71		71	66		70	60		85	81		62	59	
2.5	63	55		74	68		69	64		66	57		82	78		58	57	
3	59	51		71	64		68	59		63	54		78	76		57	55	
3.5	56	48		71	63		65	53		61	51		77	74		52	52	
4	51	43		68	62		65	52		57	49		75	73		49	50	
4.5	47	40		65	62		62	52		55	47		73	71		48	44	
**Attrition rate**	**Per 100 person-years**	**(95% CI)**		**Per 100 person-years**	**(95% CI)**		**Per 100 person-years**	**(95% CI)**		**Per 100 person-years**	**(95% CI)**		**Per 100 person-years**	**(95% CI)**		**Per 100 person-years**	**(95% CI)**	

Sex																		
Men	24.7	(22.4–27.2)		16.2	(14.4–18.2)		23.0	(19.8–26.9)		22.9	(20.3–25.9)		9.9	(8.2–11.9)		25.5	(22.2–29.2)	
Women	18.7	(17.4–20.2)		12.4	(11.3–13.6)		17.9	(15.7–20.4)		16.2	(14.5–18.1)		7.9	(6.8–9.1)		24.0	(21.7–26.6)	
**Unadjusted risk for attrition**	**HR**	**(95% CI)**	**p-value**	**HR**	**(95% CI)**	**p-value**	**HR**	**(95% CI)**	**p-value**	**HR**	**(95% CI)**	**p-value**	**HR**	**(95% CI)**	**p-value**	**HR**	**(95% CI)**	**p-value**

Sex																		
Men	Referent	—		Referent	—		Referent	—		Referent	—		Referent	—		Referent	—	
Women	0.78	(0.70–0.86)	<0.001	0.78	(0.68–0.90)	0.002	0.79	(0.71–0.88)	<0.001	0.73	(0.62–0.86)	<0.001	0.82	(0.65–1.03)	0.091	0.93	(0.78–1.10)	0.406
**Adjusted risk for attrition** †	**HR**	**(95% CI)**	**p-value**	**HR**	**(95% CI)**	**p-value**	**HR**	**(95% CI)**	**p-value**	**HR**	**(95% CI)**	**p-value**	**HR**	**(95% CI)**	**p-value**	**HR**	**(95% CI)**	**p-value**

Sex																		
Men	Referent	—		Referent	—		Referent	—		Referent	—		Referent	—		Referent	—	
Women	0.74	(0.65–0.83)	<0.001	0.85	(0.74–0.97)	0.021	0.79	(0.70–0.89)	<0.001	0.82	(0.65–1.02)	0.079	0.88	(0.66–1.18)	0.407	0.89	(0.71–1.12)	0.311

**Abbreviations:** CI = confidence interval; HR = hazard ratio.

* From death, stopping ART, or loss to follow-up.

† Multivariate analysis included only complete cases: Côte d’Ivoire (2,394), Swaziland (2,090), Mozambique (1,426), Zambia (972), Uganda (1,056), and Tanzania (938). In addition to sex, multivariate analysis adjusted for the following characteristics at ART initiation: age, CD4 count, World Health Organization HIV disease stage, and treatment regimen.
